# Neutrophils are a main source of circulating suPAR predicting outcome in critical illness

**DOI:** 10.1186/s40560-019-0381-5

**Published:** 2019-04-27

**Authors:** Hendrik Gussen, Philipp Hohlstein, Matthias Bartneck, Klaudia Theresa Warzecha, Lukas Buendgens, Tom Luedde, Christian Trautwein, Alexander Koch, Frank Tacke

**Affiliations:** 1 Department of Medicine III, RWTH-University Hospital Aachen, Aachen, 52074 Germany; 2 Department of Hepatology/Gastroenterology, Charité University Medical Center, Augustenburger Platz 1, 13353 Berlin, Germany

**Keywords:** CD87, Monocytes, Neutrophils, ICU, Organ failure, Sepsis, Prognosis, Biomarker, Innate immunity

## Abstract

**Background:**

Circulating levels of soluble urokinase plasminogen activation receptor (suPAR) have been proposed as a prognostic biomarker in patients with critical illness and sepsis. However, the origin of suPAR in sepsis has remained obscure. We investigated the potential cellular sources of suPAR by analyzing membrane-bound urokinase plasminogen activator receptor (uPAR, CD87) and evaluated its clinical relevance in critically ill patients.

**Methods:**

We studied 87 critically ill patients (44 with sepsis, 43 without sepsis) from the medical intensive care unit (ICU) in comparison to 48 standard care patients with infections and 27 healthy controls in a prospective single-center non-interventional cohort study. Cellular uPAR expression of different immune cell subsets (by flow cytometry from peripheral blood) and corresponding serum suPAR concentrations were determined upon ICU admission and at day 3. Furthermore, we analyzed the effects of serum from sepsis patients on the activation and uPAR cleavage of primary human neutrophils and macrophages in vitro.

**Results:**

In healthy controls, uPAR (CD87) expression was detected on nearly all blood neutrophils and monocytes, but only scarcely on lymphocytes. While uPAR expression on monocytes was maintained in ICU patients, only 58% of neutrophils from critically ill patients expressed uPAR, which was significantly lower than in healthy controls or standard care patients. Concomitantly, serum suPAR levels were significantly increased in ICU patients. We noted a clear inverse correlation between low neutrophilic uPAR and high serum suPAR in standard care and ICU patients, indicating that shedding of uPAR from activated neutrophils represents a main source of suPAR in systemic inflammation. Both low uPAR and high suPAR were closely associated with mortality in critically ill patients. Furthermore, serum from sepsis patients induced uPAR protein expression and subsequent receptor shedding on isolated primary neutrophils, but not on macrophages, in vitro.

**Conclusions:**

The inverse correlation between low uPAR surface expression on neutrophils and high serum suPAR in critically ill patients supports that neutrophils are a main source of shed suPAR proteins in systemic inflammation. Furthermore, high suPAR levels and low neutrophilic uPAR expression predict mortality in ICU patients.

**Electronic supplementary material:**

The online version of this article (10.1186/s40560-019-0381-5) contains supplementary material, which is available to authorized users.

## Introduction

Critically ill patients that are admitted to the intensive care unit (ICU) represent a clinically heterogeneous population with different life-threatening conditions and a large variety of underlying etiologies. Since early diagnosis and management of severely ill patients is crucial for the outcome, new diagnostic and prognostic biomarkers are urgently needed in order to assist in clinical decision-making. Despite the different etiologies and modes of injury, systemic inflammation, infections, and organ failure represent common determinants of outcome in ICU patients [1–3]. Circulating levels of the soluble plasminogen activation receptor (suPAR) are linked to inflammation, immune cell activation, and sepsis [4, 5], supporting the concept that suPAR could be a universally applicable prognostic biomarker in the setting of emergency and intensive care medicine [6, 7]. However, suPAR levels are also elevated in various clinical conditions including chronic kidney disease [8], liver cirrhosis [9], cardiac arrest [10], and HIV infection [11].

The membrane-bound form of suPAR, termed uPAR (CD87), is a glycosol-phophoatidylinositol (GPI)-anchored plasma membrane receptor [12]. Binding of ligands such as the urokinase-type plasminogen activator (uPA) activates proteases, which configure suPAR. This in turn leads to the generation of plasmin, which mediates numerous immunologic functions including migration, adhesion, angiogenesis, fibrinolysis, and cell proliferation [13, 14]. Cellular expression of uPAR has been demonstrated on activated neutrophils, monocytes, and macrophages, but also on other leukocyte subsets [15]. Subsequently, the release of suPAR into different body fluids has been viewed as the consequence of inflammatory stimulation and activation of leukocytes.

In critical illness and sepsis, serum levels of suPAR indicate an adverse prognosis, as consistently demonstrated in several cohort studies as well as a meta-analysis [4, 5, 16–18]. However, suPAR has in the ICU setting a rather low accuracy in diagnosing sepsis [16, 18, 19]. Instead, the prognostic value in critically ill patients, regardless of sepsis, seems to be associated with the presence of organ (e.g., kidney, liver, respiratory) failure [6, 16, 18–20]. These findings raise the question about the cellular source of suPAR in critically illness. We therefore comprehensively analyzed CD87 (uPAR) expression levels on peripheral blood leukocyte populations in order to identify possible variations of uPAR expression on immune cells subpopulations as well as its association with serum suPAR in critically ill patients. Furthermore, we analyzed correlations between cellular uPAR expression and clinical outcomes of ICU patients. Our data support that neutrophils are the main source of shed suPAR proteins in systemic inflammation, which is predictive for ICU mortality and acute kidney failure in critically ill patients.

## Materials and methods

### Patients and controls

This study was approved by the local ethics committee of the University Hospital Aachen, RWTH Aachen, and written informed consent was obtained from every participant or authorized relatives, in case of loss of consciousness (reference number EK 150/06). We consecutively included critically ill patients, with or without sepsis, upon admission to the medical intensive care unit of the Department of Medicine III at the RWTH University Hospital Aachen between October 2013 and March 2015, following an established protocol [21]. Septic ICU patients had a clinically suspected or verified infection diagnosed by the intensive care physicians and were subsequently treated with antibiotics. Definition of sepsis was established by diagnosed infection and an increase in the SOFA-score of greater or equal than two points [22]. Non-critically ill patients, admitted due to infectious diseases to the standard care ward, served as a diseased control population. Those patients were admitted to the hospital due to an infection diagnosed by the treating physician (based on clinical judgment, laboratory results, and/or microbial cultures) and received antibiotic therapy [21]. Of relevance for this study, acute kidney injury (AKI) was retrospectively defined for all ICU patients following the criteria proposed by the Acute Kidney Injury Network (AKIN), considering serum creatinine and urine output within the first 24 h at the ICU [20]. As a diseased control population, patients with infectious diseases admitted to the standard care ward were studied. In parallel, healthy volunteers were recruited from the local blood transfusion institute.

Blood samples were obtained by venipuncture, central venous catheter, or a permanent arterial catheter at admission and, in case of ICU patients, again 48 h after admission. Clotting was prevented by adding 250 units of heparin per milliliter of blood (Rotexmedica, Frittach, Germany).

### Isolation of peripheral blood mononuclear cells and polymorphonuclear cells

Isolation of peripheral blood mononuclear cells (PBMC) was performed using Ficoll-based density gradient centrifugation with 1077 Lymphocyte Separation Medium (PAA, Pasching, Austria), following previously established protocols [23, 24]. In brief, samples were centrifuged at 1600 rpm for 40 min at 4 °C without brake. The intermediate layer containing the PBMC was carefully harvested, washed three times with phosphate-buffered saline (PBS, PAN Biotech, Aidenbach, Germany) followed by washing at 450 rcf for 10 min at 4 °C in PBS. Polymorphonuclear cells (PMNs) were isolated using 5% dextran in PBS at 37 °C for 45 min (500,000 dextran, Merck KGaA, Darmstadt, Germany). The upper phase containing leukocytes was transferred into a new tube, and osmotic lysis of red blood cells was done by incubation for 20 s in distilled water and recovery using 10x PBS (PAN Biotech, Aidenbach, Germany).

### Flow cytometry

The PBMCs and PMNs were analyzed by multicolor flow cytometry using a FACS Canto-II (BD, Heidelberg, Germany) followed by data analysis using FlowJo Software (TreeStar, Inc., Ashland, USA). All cells were stained with a monoclonal APC-conjugated mice anti-human CD87 antibody (eBioscience, San Diego, USA, clone VIM5) or IgG2b isotype as well as with CD15, CD8, and CD16 (all from BD Horizon/Pharmingen, Heidelberg Germany) and CD11b, CD14, CD56, CD3, CD4, and CD19 (all from eBioscience, San Diego, USA) [9]. Absolute cell numbers were calculated based on FACS data and automated differential white blood cell counts (WBC).

### Monocyte and neutrophil stimulation assays

Cells were counted using a hemocytometer (Neubauer improved, peqlab, Erlangen, Germany), and PBMC were incubated in RPMI1640 medium (Sigma-Aldrich, Hamburg, Germany) containing 1% Penicillin-Streptomycin (PAA, Pasching, Austria) and 1.5% of autologous serum at a concentration of 2.5 × 10^6^ cells/mL on bacterial grade Petri dishes (Greiner Bio-one, Frickenhausen, Germany). Monocytes adhered after 35 min at 37 °C non-adherent lymphocytes were removed with the supernatant by washing five times with RPMI1640 medium [25]. After 5 days of incubation in 10% autologous serum, macrophages (of eight healthy donors) were subsequently cultivated for 48 h in RPMI1640 medium with either 10% autologous serum of *n* = 4 healthy patients or 10% serum pooled from *n* = 4 sepsis patients. As a positive control, cells were incubated in autologous serum for 6 days and subsequently stimulated with lipopolysaccharide (LPS, 1 μg/mL, Sigma-Aldrich, Hamburg, Germany) for 24 h. PMNs (of eight healthy donors) were incubated for 8 h either with 10% serum from healthy volunteers (control), interleukin-8 (IL8, 100 ng/ml, in addition to 10% healthy human serum), macrophage supernatant (MP, positive control), or 10% sepsis serum (pooled from *n* = 4 sepsis patients). Subsequently, uPAR expression of the incubated cells was analyzed by flow cytometry as described above. Dead cells were identified and excluded by Hoechst 33258 dye (Sigma-Aldrich, St. Louis, USA).

### SuPAR and cytokine quantification

The concentration of suPAR was assessed from serum or supernatant by enzyme-linked immunosorbent assay (ELISA, Virogates, Denmark), according to manufacturer’s instructions.

### Statistical analysis

Data are displayed as median and range due to the skewed distribution of the parameters. Differences between two groups were assessed by Mann-Whitney *U* test. Differences between multiple groups were assessed using the Kruskal-Wallis test (ANOVA) with Dunn’s Multiple Comparison post-test for all columns. To illustrate differences between subgroups, box plot graphics were used with whiskers ranging from minimum to the maximum value. Outliers are displayed as separate points. Correlations between variables were assessed with Spearman rank correlation tests. Kaplan-Meier curves were used to illustrate differences in survival. The optimal cut-off values achieving highest sensitivity and specificity (for uPAR and suPAR) were determined by the Youden index [26]. Receiver operating characteristic (ROC) curve analysis and the derived area under the curve (AUC) statistics were generated by plotting sensitivity against 1-specificity [27]. Statistical analysis was performed with SPSS (IBM Corp., Armonk, USA) and GraphPad Prism 5 (GraphPad Software, Inc., La Jolla, USA).

## Results

### Critically ill patients are characterized by significantly reduced uPAR expression on neutrophils

**Fig. 1:**
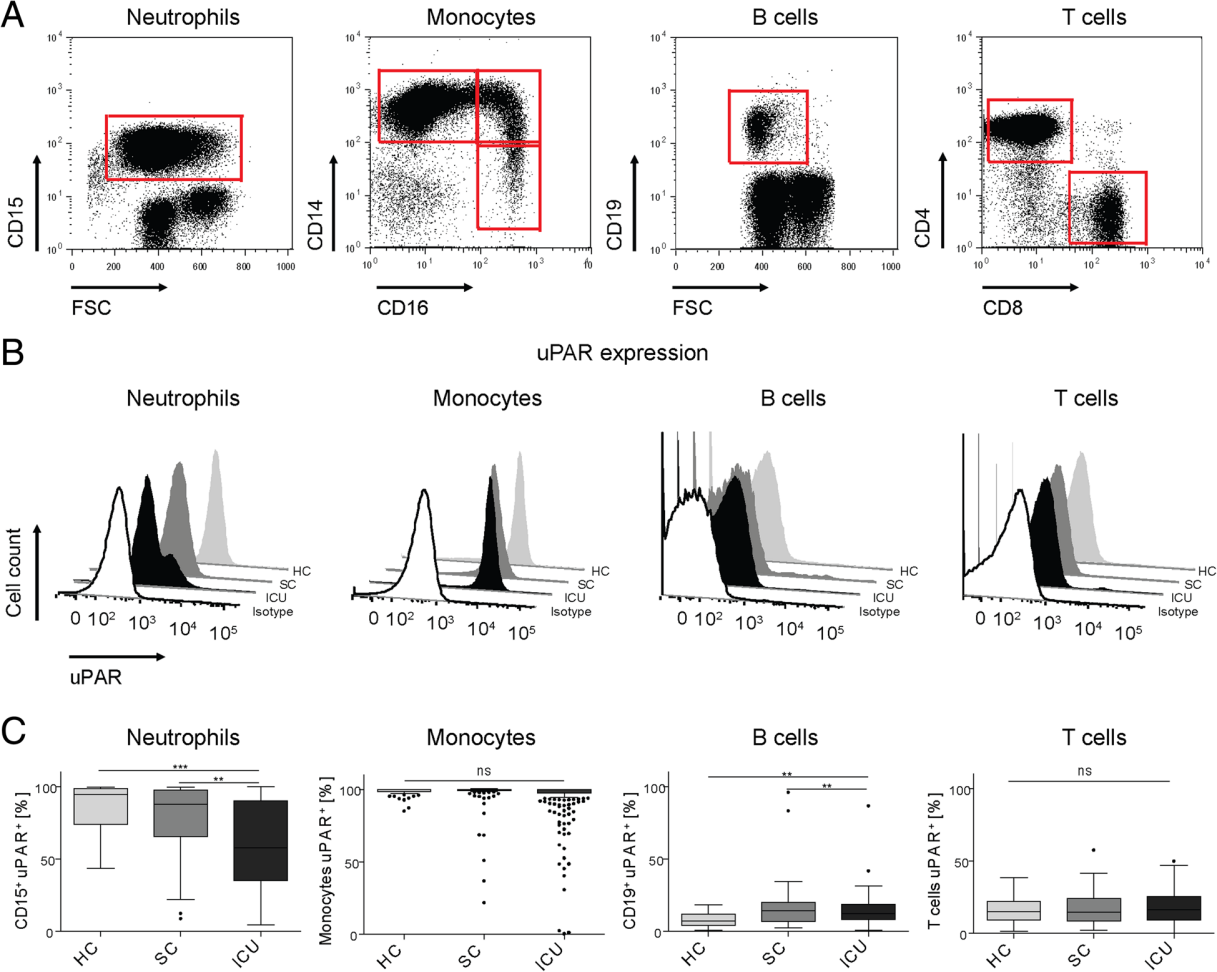
Expression of urokinase receptor (uPAR) by circulating immune cells. Immune cells were isolated from peripheral blood of 27 healthy volunteers (HC), 48 standard care patients with bacterial infections (SC) as well as 87 intensive care patients (ICU) and analyzed using multicolor flow cytometry. **a** Neutrophils were characterized as CD15^**+**^ cells, monocytes by expression of CD14^+^ and/or CD16^+^, B cells as CD19^**+**^, and T cells as CD3^**+**^ CD56^**−**^ cells. **b** The expression of uPAR (CD87) by immune cell subsets was assessed using multicolor flow cytometry. Representative histograms are shown, the white histogram shows isotype control staining. **c** Quantification of uPAR expression on immune cell subsets, based on % of positive cells by flow cytometry. One-way ANOVA and Kruskal-Wallis test (non-parametric) with Dunn’s multiple comparison test were done to compare groups. Significant differences are indicated by *p < 0.05, **p < 0.01, and ***p < 0.001

The cellular source of circulating suPAR in infectious diseases and in critical illness has remained obscure. Therefore, we comprehensively studied circulating immune cell populations in healthy controls (HC, *n* = 27), standard care (SC) patients with infectious diseases (*n* = 48), and critically ill ICU patients (*n* = 87) by flow cytometry, which allows assessing numbers and activation markers of neutrophils, monocytes, lymphocytes, and the respective cell subsets (Table 1, and Fig. 1a). In healthy volunteers, almost all neutrophils and monocytes expressed uPAR (CD87), while only small fractions of lymphocytes such as B or T cells expressed (low) levels of uPAR (Fig. 1b). While there was a non-significant trend towards lower uPAR expression on neutrophils in SC patients with infections, neutrophilic uPAR expression was significantly reduced in ICU patients (Fig. 1c). Monocytes maintained uPAR expression in SC and ICU patients as compared to controls, and a higher fraction of B cells stained positive for uPAR in ICU patients (Fig. 1c).

**Table 1:** Characteristics of the different study cohorts comprising healthy volunteers (HC), standard care patients with bacterial infections (SC), and intensive care unit (ICU) patients

	HC	SC	ICU	ICU, sepsis	ICU, no sepsis
Number of patients	27	48	87	44	43
Male/female, *n*	12/15	33/15	55/32	24/20	31/12
Age median (range), years	33 (22–77)	65.5 (21–90)	65 (18–97)	68 (18–97)	58 (23–92)
Days in hospital, median (range)	–	6.0 (2.0–25.0)	12.0 (1.0–97.0)	11.5 (1.0–97.0)	12.0 (2.0–89.0)
Days on ICU, median (range)	–	–	5.0 (1.0–79.0)	5.0 (1.0–79.0)	5.0 (1.0–37.0)
Death on ICU, *n* (%)	–	–	25 (28.7)	15 (34.1)	10 (23.3)
APACHE II score, median (range)	–	–	25 (4–46)	26 (12–41)	23 (4–46)
Absence of shock, *n* (%)	–	–	56 (64)	31 (70.5)	25 (58.1)
Leukocytes median (range), × 10^3^/μl	5.8 (3.8–8.9)	9.3 (2.1–23)	14.1 (0.5–41.9)	15.7 (0.5–42.9)	10.6 (2.7–31.4)
WBC Neutrophils median (range), %	56.4 (37.7–68.0)	78.4 (18.0–98.0)	87.7 (16.0–97.0)	90.3 (16.0–97.0)	86.0 (57.9–94.9)
WBC Monocytes median (range), %	7.2 (3.0–12.8)	7.0 (0.1–51.0)	4.7 (0.0–18.0)	4.1 (0.0–12.2)	6.4 (0.1–18.0)
WBC Lymphocytes median (range), %	32.2 (18.2–49.0)	11.6 (1.0–39.4)	4.7 (0.0–48.0)	3.8 (0.1–48.0)	6.6 (0.0–31.2)
CRP median (range), mg/l	–	86.8 (0.8–341.5)	66.6 (0.5–350.0)	135.5 (2.0–350.0)	37.4 (0.5–231.0)
PCT median (range), μg/l	–	–	1.0 (0.1–100.0)	2.18 (0.1–100.0)	0.8 (0.4–68.3)
ALT median (range), U/L	–	21.0 (5.0–84.0)	34.5 (5.0–9079.0)	26.0 (5.0–1280)	44.0 (6.0–9079.0)
AST median (range), U/L	–	32.0 (15.0–168.0)	53.5 (7.0–19,969.0)	36.0 (7.0–1178)	76.0 (14.0–19,969.0)
Gamma GT median (range) U/L	–	49.0 (8.0–636.0)	81.0 (8.0–168.0)	67.0 (4.0–781.0)	84.0 (9.0–1923)
ALP median (range), U/L	–	84.0 (35.0–286.0)	106.0 (19.0–530.0)	102.0 (39.0–350.0)	115.5 (19.0–530.0)
Bilirubin (total), mg/dl	–	0.64 (0.15–7.91)	0.78 (0.14–27.36)	0.64 (0.14–17.0)	1.0 (0.21–27.4)
Hb median (range), mmol/l	14.4 (12.7–16.0)	12.45 (7.1–17.5)	10.2 (5.6–16.1)	9.9 (7.0–16.1)	10.6 (5.6–15.5)
Creatinine median (range), mg/dl	–	0.96 (0.53–5.79)	1.5 (0.24–6.0)	1.6 (0.24–6.0)	1.9 (0.4–5.88)
GFR (range), ml/min		82.4 (8.5–139.8)	45.0 (6.0–167.4)	35.2 (6.0–130.6)	52.9 (7.3–167.4)
Urea (range), mg/dl	–	39.0 (11.0–182.0)	64.0 (10.0–233.0)	82.0 (26.0–233.0)	60.0 (10.0–213.0)
suPAR median (range), ng/ml	2.14 (0.0–3.5)	5.9 (2.1–24.1)	9.7 (0.38–38)	10.82 (0.38–38)	8.32 (1.54–38)
uPAR positive neutrophils median (range), % of neutrophils	94.7 (43.7–99.8)	87.7 (8.83–99.6)	58.3 (3.65–99.87)	51.6 (4.62–99.85)	60.9 (10.0–99.0)

*Abbreviations*: *ALP* alkaline phosphatase, *ALT/AST* alanine/aspartate aminotransferase, *APACHE II* Acute Physiology And Chronic Health Evaluation, *CRP* C-reactive protein, *GFR* glomerular filtration rate, *Hb* hemoglobin, *HC* healthy control, *ICU* intensive care unit, *SC* standard care, *PCT* procalcitonin, (*s*)*UPAR* (soluble) urokinase plasminogen activation receptor, *WBC* white blood cell count

The two right columns differentiate characteristics of ICU patients with or without sepsis

Interestingly, CD87 expression on neutrophils from ICU patients revealed two populations (two peaks in the histogram in Fig. 1b), suggesting the co-existence of high and low uPAR-expressing neutrophils specifically in critical illness (Additional file 1: Figure S1A). In order to analyze whether CD87 expression reflected the maturation stage of neutrophils, immature and mature neutrophils were separated by on low and high Forward Scatter (FSC) profile [28]. However, neutrophils with either low or high FSC characteristics displayed the same CD87 expression levels (Additional file 1: Figure S1B). Furthermore, there was no correlation between CD87 expression and the maturation marker CD11b on neutrophils (*r* = 0.100, *p* = 0.204).

### Neutrophilic uPAR expression and circulating suPAR are inversely correlated in critical illness

**Fig. 2:**
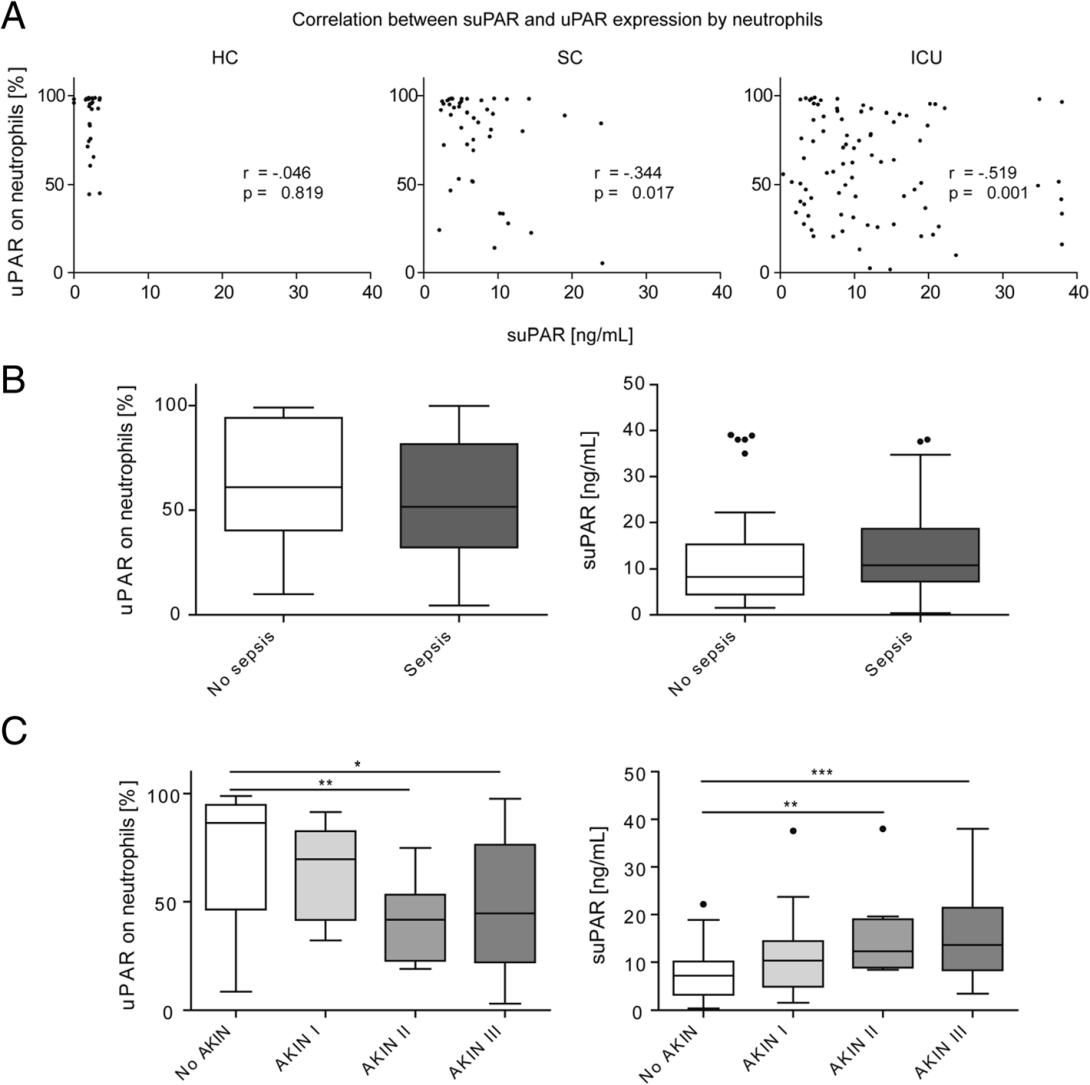
Association between neutrophilic uPAR expression and circulating suPAR in critically ill patients. Flow-cytometric expression of uPAR (CD87) on circulating neutrophils and serum levels of suPAR were analyzed in 27 healthy volunteers, 48 standard care patients with bacterial infections, and 87 intensive care patients at baseline. **a** Correlation analysis of uPAR expressed on neutrophils and suPAR in peripheral blood in the different cohorts. Correlation coefficient (*r*) and *p* values are based on Spearman rank correlation test and given in the figure. **b** uPAR expression by neutrophils and suPAR serum concentrations in septic and non-septic ICU patients. **c** uPAR expression by neutrophils and suPAR serum concentrations in ICU patients, dependent on the stage of acute kidney injury (AKI) as defined by the Acute Kidney Injury Network (AKIN). Significant differences are indicated by *p < 0.05, **p < 0.01, and ***p < 0.001

While neutrophilic expression of CD87 decreased in ICU patients compared to SC or HC, suPAR serum levels progressively increased between HC, SC, and ICU patients (Table 1), in line with prior reports [4, 16]. We found a clear inverse correlation between uPAR on neutrophils and suPAR in SC (*r* = − 0.344, *p* = 0.017) and ICU patients (*r* = − 0.519, *p* < 0.001; Fig. 2a), supporting the hypothesis that circulating suPAR in systemic inflammation is primarily the result of uPAR cleavage from neutrophils. Interestingly, neither neutrophilic uPAR nor suPAR differed significantly between ICU patients with sepsis (*n* = 44) as compared to non-sepsis (*n* = 43), in line with prior findings [16], although there was a trend towards reduced neutrophilic uPAR and higher serum suPAR in sepsis (Fig. 2b). The detailed disease etiologies leading to ICU admission are summarized in Table 2. Only 3 out of 87 ICU patients were neutropenic at ICU admission (defined as WBC < 1.5 × 10^3^/μl), so that our cohort is not suited to conclude whether the correlation between suPAR and neutrophilic uPAR is also present in neutropenia.

**Table 2:** Disease etiology of the study population leading to ICU admission

	Sepsis	Non-sepsis
	*n* = 44	*n* = 43
Etiology of sepsis critical illness		
Site of infection *n* (%)		
Pulmonary	21 (48%)	
Abdominal	14 (32%)	
Urogenital	6 (14%)	
Other	3 (6%)	
Etiology of non-sepsis critical illness *n* (%)		
Cardiopulmonary disorder		10 (23%)
Acute pancreatitis		6 (14%)
Acute liver failure		3 (7%)
Decompensated liver cirrhosis		4 (9%)
Severe gastrointestinal hemorrhage		7 (16%)
Elektrolyte imbalance		2 (5%)
Neurological disorders		2 (5%)
Non-sepsis other		9 (21%)

Next, to reduced cleavage from neutrophils, reduced renal excretion could contribute to increased suPAR levels [8]. Serum suPAR had been linked to acute kidney injury (AKI) in surgical ICU patients [20]. In fact, we observed lower neutrophilic uPAR expression according to the stage of AKI during the first 24 h at the ICU. Reduced uPAR on neutrophils was particularly found in AKI stages II (*n* = 11, neutrophilic uPAR expression median 41.7%) and III (*n* = 28, median 44.5%), which was mirrored by significantly increased serum suPAR (AKI II, median 12.26 ng/ml; AKI III, 13.58 ng/ml) (Fig. 2c). Both suPAR and neutrophilic uPAR were correlated with the glomerular filtration rate and other parameters of renal failure (Table 3). Furthermore, suPAR and neutrophilic uPAR were associated with biomarkers of systemic inflammation (i.e., total leukocytes, C-reactive protein, procalcitonin, cytokines) as well as parameters indicating hepatic function (Table 3).

**Table 3:** Correlation analysis between serum suPAR and neutrophilic uPAR with other laboratory and clinical parameters markers in ICU patients

Parameter	suPAR (serum)		uPAR (neutrophils)	
	*r*	*p*	*r*	*p*
Inflammation				
C-reactive protein	0.232	*0.015*	− 0.264	*0.007*
Procalcitonin	0.422	< *0.001*	− 0.329	*0.001*
WBC	0.169	0.059	− 0.227	*0.017*
Interleukin-6	0.506	< *0.001*	− 0.345	*0.001*
TNF	0.253	*0.009*	− 0.24	*0.013*
Interleukin-10	0.248	*0.012*	− 0.24	*0.014*
Renal function				
Creatinine	0.512	< *0.001*	− 0.342	*0.001*
GFR (creatinine)	− 0.511	< *0.001*	0.289	*0.003*
Urea	0.469	< *0.001*	− 0.271	*0.006*
Liver function				
AST	0.3	*0.003*	− 0.214	*0.028*
ALT	0.159	0.075	− 0.121	0.137
Bilirubin	0.5	< *0.001*	− 0.238	*0.014*
Albumin	− 0.24	*0.019*	0.204	*0.04*
Gamma GT	0.227	*0.02*	− 0.19	*0.043*
Alkaline phosphatase	0.246	*0.018*	− 0.231	*0.025*
Clinical course				
Days in hospital	0.301	*0.009*	− 0.093	0.238
Days at ICU	0.21	*0.027*	− 0.03	0.391

*Abbreviations*: *ALT/AST* alanine/aspartate aminotransferase, *GFR* glomerular filtration rate, *ICU* intensive care unit, *TNF* tumor necrosis factor, (*s*)*uPAR* (soluble) urokinase plasminogen activation receptor, *WBC* white blood cell count

Spearman rank correlation test, significant correlations (*p* < 0.05) highlighted in italics

### Neutrophilic uPAR expression and circulating suPAR are associated with prognosis of critically ill patients

**Fig. 3:**
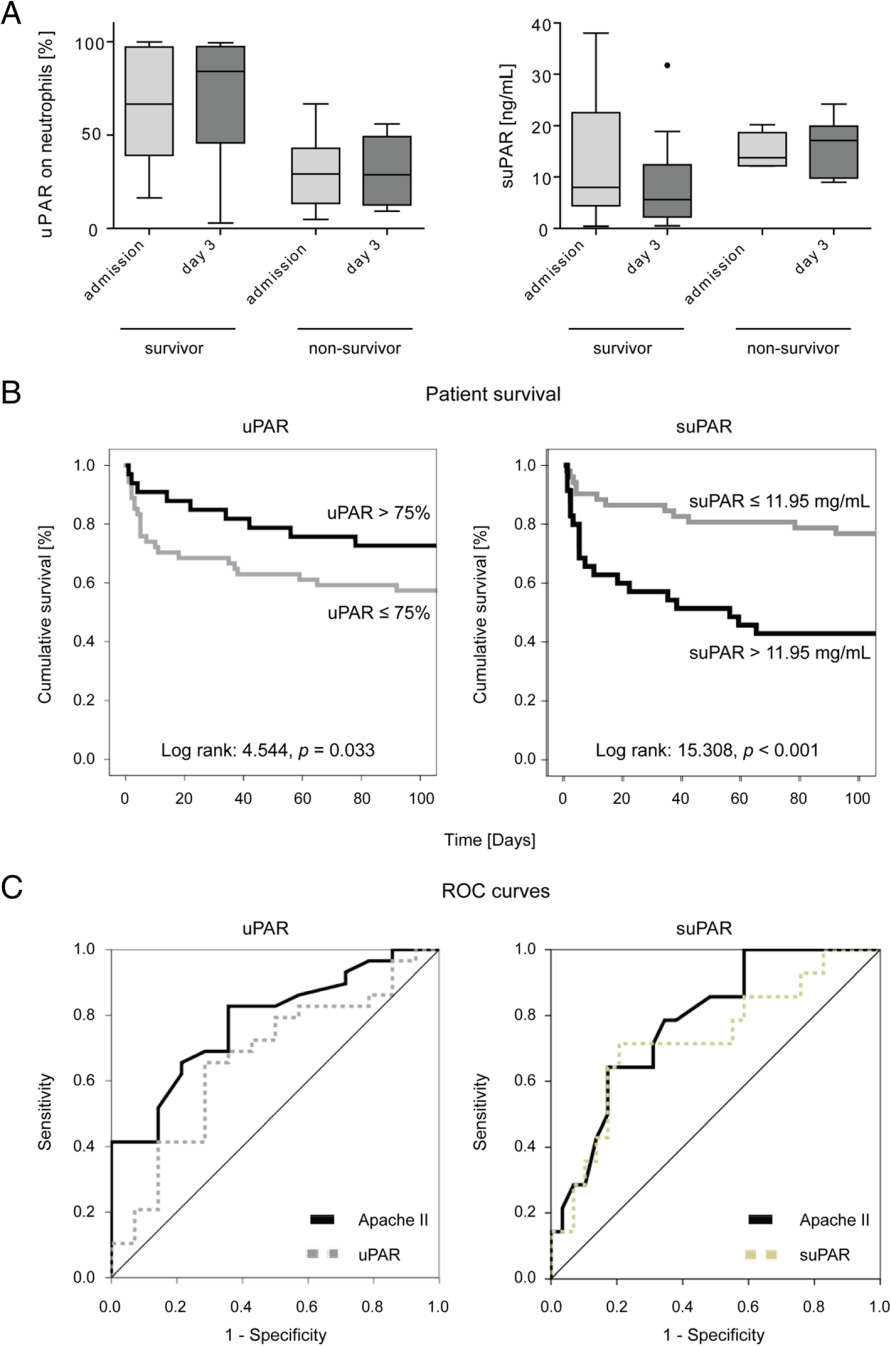
Prognostic relevance of neutrophilic uPAR expression and circulating suPAR levels. Flow-cytometric expression of uPAR (CD87) on circulating neutrophils and serum levels of suPAR were measured at admission to the ICU and 48 h later (day 3). **a** Neutrophilic uPAR expression and circulating suPAR levels in ICU patients, divided into patients that survived and patients that died at the ICU. **b** Kaplan-Meier survival curves display the different survival rates of ICU patients, depending on either high or low neutrophilic uPAR (cut-off 75%) or circulating suPAR levels (cut-off 11.95 ng/ml) at ICU admission. **c** ROC curve analyses comparing the predictive power of suPAR (AUC 0.727) and uPAR (AUC 0.665) at admission for mortality with the established clinical ICU score APACHE II (AUC 0.781). Abbreviations: APACHE Acute Physiology and Chronic Health Evaluation, AUC area under the curve, ROC receiver operating characteristics

In our cohort, 25 out of the 87 critically ill patients (28.7%) died at the ICU. Strikingly, uPAR expression on neutrophils at ICU admission were significantly lower in non-survivors (66.63% in survivors vs 29.10% in non-survivors, *p* = 0.023, Fig. 3a). Correspondingly, serum suPAR levels at ICU admission were higher in non-surviving (median 13.74 ng/ml) compared with surviving (7.94 ng/ml) individuals, although this difference did not reach statistical significance (*p =* 0.277, Fig. 3a). In survivors, there was a trend towards restoration of uPAR expression on neutrophils and reduction of suPAR at day 3 (Fig. 3a). On the contrary, non-surviving patients were characterized by persistently low neutrophilic uPAR and high (even slightly increasing) suPAR at day 3 (Fig. 3a), indicating that the recovery of uPAR on neutrophils might be beneficial in the pathogenesis of systemic inflammation.

Based on these findings, we next tested the prognostic value of uPAR expression on neutrophils as well as serum suPAR at ICU admission to predict survival. By Cox regression analysis, suPAR was indeed significantly associated with ICU mortality (*p* = 0.036), while uPAR demonstrated a similar trend (*p* = 0.170). Using Kaplan-Meier survival curve analyses with optimal cut-off values for neutrophilic uPAR (75%) or serum suPAR (11.95 ng/ml), low uPAR (≤ 75%) as well as high suPAR (> 11.95 ng/ml) clearly separated surviving and non-surviving ICU patients (Fig. 3b), emphasizing the prognostic relevance of both neutrophilic uPAR and circulating suPAR in critical illness.

As uPAR and especially suPAR could discriminate between survival and death in the ICU cohort, we tested whether the prognostic value of uPAR and suPAR were equal to established scores like APACHE II by using ROC curve analyses. Whereas APACHE II achieved the highest AUC statistics of 0.781, suPAR and uPAR reached 0.727 and 0.665 (Fig. 3c), demonstrating similar power for suPAR (as a single marker) compared to an established clinical score in predicting mortality.

### Circulating factors from sepsis patients induce uPAR expression and shedding in neutrophils

**Fig. 4:**
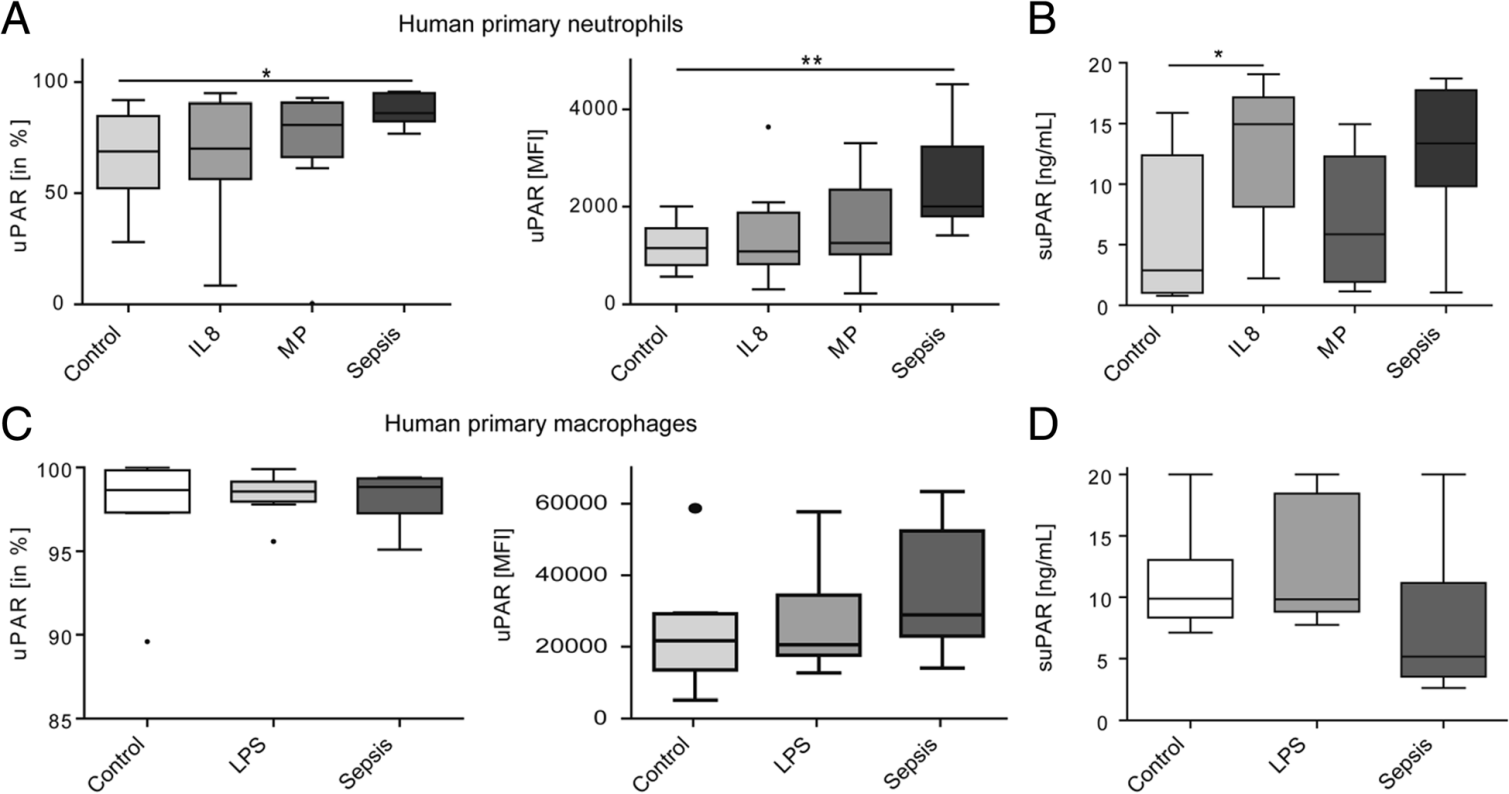
Impact of inflammatory mediators on uPAR expression of human neutrophils and monocytes ex vivo. Primary human neutrophils (**a**, **b**) and monocytes (**c**) were isolated from healthy controls (*n* = 8), and uPAR expression was analyzed by flow cytometry. **d** Human neutrophils were incubated for 8 h either with 10% serum from healthy volunteers (control), interleukin-8 (IL 8, 100 ng/ml, in addition to 10% healthy human serum), macrophage supernatant (MP), or 10% sepsis serum (pooled from *n* = 4 sepsis patients. **b** Release of suPAR was analyzed in the supernatant of the neutrophil cultures. **c** Human macrophages were cultured for 5 days in 10% serum from healthy controls and subsequently exposed to 10% serum from sepsis patients for 48 h. As a positive control, macrophages were cultured for 6 days, followed by stimulation with LPS (100 ng/ml) + 10% healthy human serum for 24 h. **d** Release of suPAR in the supernatant from macrophage cultures with the different conditions

While the clinical data from ICU patients supported the conclusion that uPAR is cleaved from the surface of primarily neutrophils, we wanted to exclude the alternative hypothesis that systemic inflammation downregulates uPAR expression on either neutrophils or monocytes. We therefore cultivated primary human neutrophils (viability 89.6%) from healthy volunteers (*n* = 8) and incubated the cells for 8 h with either 10% serum from healthy controls, IL-8 (100 ng/ml) or macrophage supernatant (to activate neutrophils) as well as with 10% serum from patients with sepsis (Fig. 4a). Serum from sepsis patients strongly induced surface uPAR expression on human neutrophils, leading to uPAR expression on almost all neutrophils as well as to a significantly higher mean fluorescence intensity (MFI) of the staining. Concomitantly, IL8 and incubation with sepsis serum increased suPAR concentration in the supernatant of neutrophil cultures, supporting that sepsis serum not only induces uPAR expression, but also uPAR shedding (i.e. suPAR release) from neutrophils (Fig. 4b).

In addition, primary human macrophages (from healthy volunteers) were cultured for 5 days in pooled human serum. Subsequently, macrophages (viability 94.6%) were either stimulated after additional 24 h with LPS (for 24 h, positive control) or cultured in the presence of pooled serum from sepsis patients for 48 h. While all primary macrophages (at day 7 in culture) stained uniformly positive for uPAR (Fig. 4c), stimulation with either LPS or 10% sepsis serum led only to a mild, non-significant increase in uPAR MFI by FACS staining on macrophages (Fig. 4c). Measuring accompanying suPAR concentrations in the supernatant cultures did not indicate that shedding was induced by sepsis serum from primary human macrophages (Fig. 4d). These data emphasize that uPAR expression reflects an inflammatory response primarily of neutrophils, indicating that neutrophilic uPAR is a major source of circulating suPAR in critical illness.

## Discussion

The soluble form of uPAR (CD87), suPAR, has emerged as an attractive biomarker in various clinical settings for several reasons [6, 29–31]. From a technical point of view, suPAR levels are relatively stable in human serum and its measurement is simple [16, 30]. From a clinical point of view, this marker has been linked to quite a range of complications and adverse prognosis in manifold disease settings [4, 8, 31], and even in seemingly healthy volunteers [32, 33]. The ability of suPAR to help in risk stratification is particularly interesting in the emergency room [34, 35] or the ICU setting [18, 36], where acutely ill patients require an early risk assessment to tailor therapeutic interventions and allocate appropriate ICU resources. However, despite its practical utility for prognostication in the ICU setting, suPAR appears to be quite non-specific as to the cause of disease, to the presence of sepsis and to the nature of concomitant organ failure(s) [30]. This raised the question about the cellular origin of circulating suPAR in critical illness.

By analyzing a cohort of 87 prospectively enrolled critically ill patients in comparison to 48 standard care patients with infections and 27 healthy controls, our study revealed the inverse correlation between CD87 (uPAR) expression on peripheral blood neutrophils and circulating suPAR in ICU patients. Moreover, primary human neutrophils upregulated uPAR upon stimulation with serum from sepsis patients, which was associated with increased shedding of the receptor into the supernatant. Collectively, our data strongly indicate that neutrophils are a main source of suPAR in critical illness in vivo.

Neutrophils contain preformed intracellular pools of uPAR that are localized mainly in azurophilic and gelatinase granules as well as secretory vesicles [37]. Within the granulocytes, CD87 is expressed on neutrophilic granulocytes at the band and segmented stage of development and is usually not expressed at earlier stages of maturation [38, 39]. Importantly, CD87 is also downregulated with neutrophilic apoptosis, thus end-stages of the cell cycle [40]. Upon activation, neutrophils translocate their preformed intracellular uPAR pool to the plasma membrane. Subsequently, the D2D3 linker region gets cleaved through proteases, leading to shedding [41]. The reduced uPAR levels on neutrophils of ICU patients in our cohort could therefore either reflect an immature state as in freshly released precursors [38], be the result of increased receptor shedding due to activation [41], or reflect “end-stage neutrophils” that had apoptotic cell death already initiated [40]. We could not identify a clear association between neutrophil maturation and CD87 expression in critically ill patients, making it most likely that uPAR shedding from neutrophils majorly contributes to high serum suPAR levels in ICU patients.

In our study, uPAR was expressed not only on neutrophils, but also on monocytes that also demonstrated a reduced uPAR surface expression in ICU patients compared to standard care patients and healthy controls. Our in vitro data indicate that systemic mediators in sepsis patients induce uPAR shedding on neutrophils, but not macrophages. Although neutrophils appeared to represent the major source of suPAR in ICU patients in vivo, suPAR might be released from other cells, including non-circulating cells, as well. This would, for instance, explain why suPAR serum levels can be elevated in neutropenic patients with infections [42]. In fact, the cellular origin of suPAR may substantially differ in organ-specific disease settings, and such mechanisms may additionally contribute to circulating suPAR in ICU patients with organ failure(s).

The pathogenic involvement of suPAR has been best studied in focal segmental glomerulosclerosis (FSGS), which is associated with segmental sclerosis in glomeruli, proteinuria, and, ultimately, progressive renal failure [43]. SuPAR acts as a tripartite complex of suPAR, APOL1 risk variants, and αvβ3 integrin on podocytes, resulting in podocyte foot process effacement, disrupted glomerular barrier function, proteinuria, and thereby chronic kidney disease [44]. Using mouse models of FSGS, bone marrow-derived immature myeloid cells were identified as a main source of circulating suPAR contributing to proteinuric kidney disease [45]. In patients with liver diseases, non-classical monocytes (expressing CD14 and CD16) have been suggested as the main cellular source of suPAR in the setting of acute liver failure [11]. However, human primary monocyte-derived macrophages responded relatively poorly to stimulation with sepsis serum regarding their uPAR expression or shedding in the in vitro experiments of our study, suggesting that these sources of myeloid cells appear less relevant in critical illness.

In our study, we did not find significant differences in either uPAR or suPAR expression between sepsis and non-sepsis patients. While this finding is in agreement with prior studies [16, 17], it is a bit surprising, given the higher neutrophil numbers in patients with sepsis than without sepsis. Functional analyses using urokinase receptor (CD87) deficient mice demonstrated that that uPAR is crucially involved in the host defense against sepsis caused by the Gram-negative bacterium *Burkholderia pseudomallei* [46] as well as against severe malaria [47]. Further studies are needed to assess whether removing suPAR from the circulation, for instance by hemoadsorption or plasma exchange [48], could hold therapeutic value in patients with sepsis.

In line with prior studies [4, 16–18, 36], high suPAR and correspondingly low neutrophilic uPAR levels were both associated with mortality in critically ill patients. Interestingly, serum suPAR, but not neutrophilic uPAR, reached a precision in predicting mortality (as a single biomarker) comparable to the composite APACHE-II score. While our study thereby confirms the value of suPAR as a prognostic biomarker, the identification of the cellular source by our work should inform future studies that may focus on the pathogenic involvement of this pathway for adverse outcome. It has already been demonstrated in non-ICU settings that dynamic changes in suPAR are indicative of effective responses to therapeutic interventions [33]. Based on the strikingly different uPAR levels on neutrophils during the first 3 days of ICU treatment in surviving vs non-surviving patients in our study, restoring neutrophil function, as reflected by high uPAR expression, could be an interesting surrogate for myeloid-specific interventions.

## Conclusions

The soluble form of the urokinase plasminogen activator receptor (uPAR), termed suPAR, has been described as a prognostic serum biomarker in various disease settings. By analyzing a cohort of 87 prospectively enrolled critically ill patients in comparison to 48 standard care patients with infections and 27 healthy controls, we herein demonstrate that suPAR levels in ICU patients are inversely correlated with uPAR (CD87) expression on neutrophils. Moreover, low neutrophilic uPAR expression by FACS and high suPAR in serum are characteristic to critical illness, are independent from sepsis, are associated with acute kidney injury, and predict mortality in ICU patients. Accompanying in vitro experiments confirmed that uPAR is induced in primary human neutrophils, but not in primary human macrophages, by circulating mediators found in serum of sepsis patients. Our data thereby demonstrate that neutrophils are the main cellular source of suPAR in conditions of critical illness, which helps to better understand the value of this biomarker and may guide future therapeutic studies aiming at interfering with this pathway.

## Additional file

Additional file 1: **Figure S1.** Expression of CD87 on neutrophils. (DOCX 99 kb)

## Abbreviations

AKI: Acute kidney injury
APACHE-II: Acute Physiology and Chronic Health Evaluation
CRP: C-reactive protein
FACS: Fluorescence activated cell sorting
GFR: Glomerular filtration rate
HC: Healthy controls
ICU: Intensive care unit
IL: Interleukin
SC: Standard care
SOFA: sequential organ failure assessment
suPAR: Soluble urokinase plasminogen activation receptor
TNF: Tumor necrosis factor
uPAR: Urokinase plasminogen activation receptor
WBC: White blood cell count
