# Correction to: Neutrophils are a main source of circulating suPAR predicting outcome in critical illness

**DOI:** 10.1186/s40560-019-0397-x

**Published:** 2019-08-02

**Authors:** Hendrik Gussen, Philipp Hohlstein, Matthias Bartneck, Klaudia Theresa Warzecha, Lukas Buendgens, Tom Luedde, Christian Trautwein, Alexander Koch, Frank Tacke

**Affiliations:** 1 Department of Medicine III, RWTH-University Hospital Aachen, 52074 Aachen, Germany; 2 Department of Hepatology/Gastroenterology, Charité University Medical Center, Augustenburger, Platz 1, 13353 Berlin, Germany

**Correction to: J Intensive Care (2019) 7:26**

**https://10.1186/s40560-019-0381-5**

In the original publication of this article [1], the content of Authors’ contributions needs to be revised as below:

HG and PH collected blood samples, performed FACS analyses, and conducted data analyses. MB and KTW supported the in vitro experiments. LB, TL, and CT contributed to data collection and analysis. AK and FT designed and supervised the study. HG drafted the manuscript and FT made the final corrections. All authors corrected and approved the manuscript.
