# Supplementary material for: Neutrophils are a main source of circulating suPAR predicting outcome in critical illness
Source: J Intensive Care. 2019 Apr 27;7:26. doi: 10.1186/s40560-019-0381-5 (PMC6487050; doi:10.1186/s40560-019-0381-5)
Supplement: Supplementary file 1 — Figure S1. Expression of CD87 on neutrophils. (DOCX 99 kb) [file 40560_2019_381_MOESM1_ESM.docx]

**Supporting Figure**


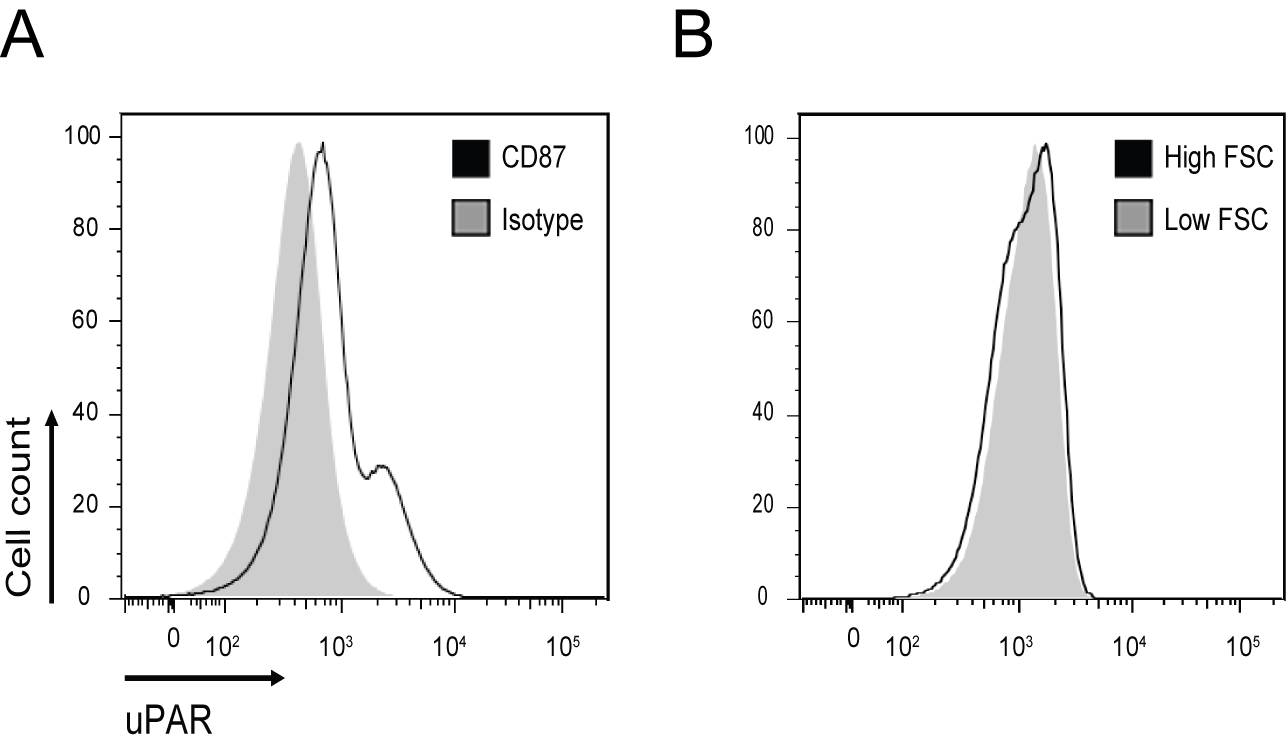


**Figure S1: Expression of CD87 on neutrophils.** (A) The expression of uPAR (CD87) by immune neutrophils was assessed using multicolor flow cytometry. Representative histograms are shown for critically ill patients. Overlay of isotype control (grey) and uPAR (black line) reveals that the majority of neutrophils in ICU patients have low level uPAR expression (shift of histograms), while a small fraction of neutrophils expresses high levels of uPAR. (B) For all cohorts, neutrophils were separated based on the forward scatter (FSC) profile, reflecting different cell sizes. Representative histograms are shown, the black line indicates high FSC cells, the grey plot shows low FSC cells. Neutrophils with high or low FSC do not differ in their uPAR (CD87) expression.
